# Quantification of 6-Mercaptopurine and Its Metabolites in Patients with Acute Lympoblastic Leukemia Using Dried Blood Spots and UPLC-MS/MS

**DOI:** 10.3390/scipharm86020018

**Published:** 2018-04-25

**Authors:** Supandi Supandi, Yahdiana Harahap, Harmita Harmita, Rizka Andalusia

**Affiliations:** 1 Department of Pharmacy, Faculty of Pharmacy and Science, Universitas Muhammadiyah Prof. Dr. HAMKA, Jakarta 13460, Indonesia; supandi@uhamka.ac.id; 2 Faculty of Pharmacy, Universitas Indonesia, Kampus UI Depok, West Java 16424, Indonesia; harmita@ui.ac.id; 3 Department of Research and Development of Dharmais Cancer Hospital, Jakarta 11420, Indonesia; rizka_andalusia@yahoo.com

**Keywords:** 6-mercaptopurine, dried blood spots, acute lymphoblastic leukemia

## Abstract

This research aimed to quantitatively bioanalyze 6-mercaptopurine (6-MP), 6-methylmercaptopurine (6-MMP), and 6-thioguanosine-5′-monophosphate (6-TGMP) in dried blood spots (DBS) prepared from a small volume of acute lymphoblastic leukemia (ALL) patients. Analytes on the DBS card were extracted using 90% methanol with 5-fluorouracil (5-FU) as an internal standard. Analytical separation was performed on a Waters Acquity^®^ UPLC BEH AMIDA column of 1.7 μm (2.1 × 100 mm) with a mobile phase mixture of 0.2% formic acid in water and 0.1% formic acid in acetonitrile-methanol, with gradient elution and a flow rate of 0.2 mL/min. Mass detection of 6-MP, 6-MMP, 6-TGMP, and 5-FU showed *m*/*z* values of 153.09 > 119.09, 167.17 > 126.03, 380.16 > 168.00, and 129.09 > 42.05, respectively. This DBS method had a run time of 5 min and yielded a linear calibration curve over a range of 25.5–1020 ng/mL for 6-MP, 6-MMP, and 6-TGMP. Analyte analysis in 22 of 24 ALL patients showed that the measured value of 6-TGMP as an active metabolite was in the range of 29–429 pmol/8 × 10^8^ erythrocytes. Five of 22 patients had concentrations in a therapeutic range, which indicates that the treatment is effective, while 17 of 24 patients had concentrations below the therapeutic range, which indicates that a treatment dose adjustment is needed. The measured value of 6-MMP, an inactive metabolite, was in the range of 28–499 pmol/8 × 10^8^ erythrocytes, which includes concentrations below the hepatotoxic range. The method employed here can thus be effectively utilized to support therapeutic drug monitoring.

## 1. Introduction

**Figure 1:**
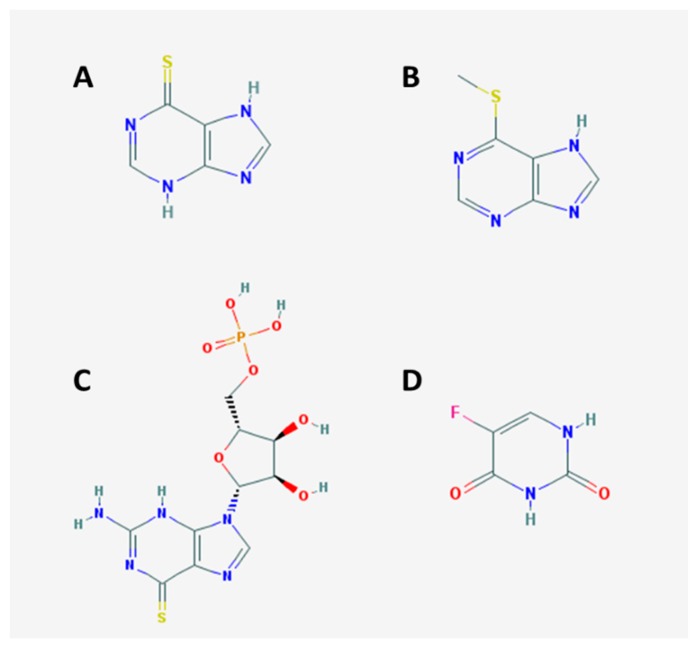
Chemical structures of (**A**) 6-mercaptopurine (6-MP); (**B**) 6-methylmercaptopurine (6-MMP); (**C**) 6-thioguanosine-5′-monophosphate (6-TGMP); and (**D**) 5-fluorouracil (5-FU).

Acute lymphoblastic leukemia (ALL) is a malignant (clonal) disease where early lymphoid precursors proliferate and replace normal hematopoietic cells in bone marrow [1]. The chemotherapeutic agent 6-mercaptopurine (6-MP; Figure 1A) is used in most treatment protocols of ALL [2]. 6-Mercaptopurine is an antineoplastic drug that belongs to the class of antimetabolites. It is an inactive prodrug that goes through three metabolic pathways: The first is through the enzyme hypoxanthine-guanine phosphoribosyltransferase (HGPRT), then into thioinosine monophosphate (TIMP) and, subsequently, into 6-thioxanthosine-5-monophosphate (TXMP) and active metabolite 6-thioguanosine nucleotides (mono-, di-, and triphosphate). 6-Thioguanosine nucleotides (TGN) are active metabolites that incorporate with DNA and induce the breaking of DNA chains. The second pathway is through the enzyme S-thiopurine methyltransferase (TPMT) to form 6-methylmercaptopurine (6-MMP; Figure 1B), and the third pathway is through the enzyme xanthine dehydrogenase to form 6-thiouric acid (6-TU), 6-MMP, and 6-TU, which are inactive metabolites [2,3,4]. High active metabolite levels have been correlated with good therapeutic efficacy and hematology toxicity, whereas high inactive metabolite levels are associated with hepatotoxicity [5]. 6-Mercaptopurine also displays a range of possible adverse drug reactions and a narrow therapeutic index; hence, the therapeutic index for each individual needs to be monitored [6]. 

Therapeutic drug monitoring (TDM) is a clinical practice for measuring specific drug levels at designated intervals to ensure that a constant concentration in a patient’s bloodstream is maintained and thus that individual dosage regiments can be optimized. Blood concentration in the maximum concentration phase and elimination phase determines the effectiveness of a treatment [7]. 6-MP drug levels are monitored mostly via venipuncture [3,5,6,8]. The disadvantages of venous blood sampling, one means of venipuncture, are that it requires large blood samples and, for invasive processes, a phlebotomist. 

Dried blood spots (DBS) represent a bio-sampling method that has been developed recently for therapeutic drug monitoring [9]. The main benefits of DBS include a potential for long storage periods at room temperature, convenient transport in envelopes, and minimally-invasive blood sampling without direct sample processing. Since the burden of DBS methods is so low, they are particularly suitable for the elderly, children, and infants, for whom venipuncture is problematic [10,11,12]. Previous studies have reported analysis, validation, and applications of 6-MP and 6-MMP on childhood leukemia patients [13,14]. However, use of the DBS method based on analysis of 6-MP, 6-MMP, and 6-TGMP, with a larger number of patients, has not been reported. Here, in order to provide a reliable therapeutic drug monitoring method, 6-MP was analyzed in DBS from children with acute lymphoblastic leukemia in the maintenance phase at Dharmais Cancer Hospital. 

## 2. Materials and Methods

### 2.1. Reagents and Chemicals

6-Mercaptopurine, 6-methylmercaptopurine and 5-fluorouracil were acquired from Sigma-Aldrich (Saint Louis, MO, USA). 6-Thioguanosine-5′-monoposphate (6-TGMP; Figure 1C) was acquired from Jena Bioscience (Löbstedter, Jena, Germany). Acetonitril and methanol high performance liquid chromatography (HPLC)-grade from Merck (Kenilworth, NJ, USA). All water was HPLC-grade and prepared using a Millipore Direct-QTM 5 water system (Millipore, Watford, Hertfordshire, UK). Whole blood was acquired from the Indonesian Red Cross.

### 2.2. Apparatus and Analytical Conditions

Chromatographic analysis was performed using a Waters Acquity^®^ UPLC bridged ethylene hybrid AMIDA column of 1.7 μm (2.1 × 100 mm) (Waters, Milford, MA, USA). The mobile phases were 0.2% formic acid in water and 0.1% formic acid in acetonitrile–methanol. The flow rate was 0.2 mL/min under gradient elution conditions. The run time was approximately 5.0 min. The mass selective detector in electrospray ionization (ESI) operated in positive mode for analytes and in negative mode for 5-fluorouracil (5-FU; Figure 1D) as an internal standard (IS). Mass spectrometric detection was performed on a Waters Xevo TQD Triple Quadrupole (Waters, Milford, MA, USA). Multiple reaction monitoring was employed with wide mass resolutions for MS1 and wider mass resolutions for MS2. High-purity nitrogen was used as a source and collision gas. The MS was operated for both and multiple reaction monitoring mode.

### 2.3. Method Validation in Dried Blood Spots

The method validation process in this study was performed in accordance with the European Medicines Agency (EMEA) guidelines of bioanalytical method validation [15]. The full validation analytical method in dried blood spots was conducted in terms of parameters, selectivity, carry-over, lower limit of quantification (LLOQ), linear calibration curve, accuracy, precision, the matrix effect, dilution integrity, and stability parameters.

### 2.4. Application of the Method

The ALL patients from whom DBS samples were taken fulfilled the following inclusion criteria:

- The ALL was in the maintenance phase;
- The patient received 6-MP as per a therapeutic protocol and in a steady state;
- The patient was 0–18 years old during blood collection; and
- The patient was without kidney or liver disorders.

Blood samples of up to 100 μL were collected at least 8 h after the preceding 6-MP dose (75 mg/m^2^/day). The blood was spotted on DBS paper and dried for 3 h. DBS paper was then inserted into a zip-lock bag and stored at room temperature until analysis was conducted.

### 2.5. Sample Preparation

The spot sample was cut and placed into a tube. Extraction solution consisted of 1 mL of 90% methanol with 100 µL of internal standard 5-FU. Tubes were sonicated for 25 min at 50 °C. The supernatant was later transferred into test tubes and evaporated with nitrogen for 25 min at 40 °C. The mixture reconstituted in 100 µL of acetonitrile 10% and was then vortexed and sonicated for 10 s. It was then centrifuged at 3000 rpm for 10 min, and an aliquot was injected into the chromatographic system. 

This research was approved by an ethics committee at Dharmais Cancer Hospital (No: 019/KEPK/IV/2016).

## 3. Results and Discussion

### 3.1. Method Development and Optimization

**Figure 2:**
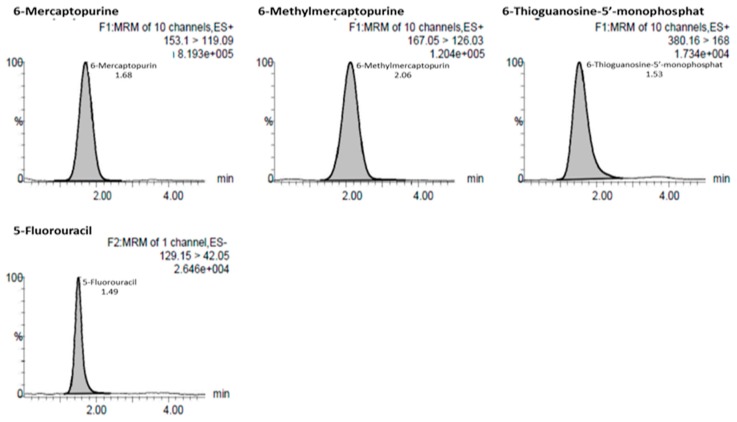
Chromatograms of 6-mercaptopurine, 6-methylmercaptopurine, 6-thioguanosine-5′-monophosphate, and 5-fluorouracil.

Mass detection was performed using Waters Xevo TQD with positive ESI for 6-MP, 6-MMP, and 6-TGMP, and negative ESI for 5-FU in multiple reaction monitoring mode. Detection of 6-MP, 6-MMP, 6-TGMP, and 5-FU showed *m*/*z* values of 153.09 > 119.09, 167.17 > 126.03, 380.16 > 168.00, and 129.09 > 42.05, respectively. The mobile phases were 0.2% formic acid in water and 0.1% formic acid in acetonitrile-methanol. The flow rate was 0.2 mL/min under gradient elution conditions, and the run time was approximately 5.0 min. Representative chromatograms of the finalized chromatographic conditions, showing 6-MP, 6-MMP, 6-TGMP, and 5-FU, are depicted in Figure 2.

### 3.2. Method Validation

Simultaneous DBS analysis of 6-MP, 6-MMP, and 6-TGMP in vitro was valid. All parameters fulfilled the acceptance criteria of the EMEA Bioanalytical Method Validation Guideline [16]. 

#### 3.2.1. Selectivity

No interference or extra peak was observed in endogen compounds, which were investigated by analyzing six different sources (hematocrit and blood type). The selectivity of the method was determined by quantifying analytes at LLOQ concentrations. Absence of interfering components is acceptable where the response is less than 20% of the LLOQ for analytes and the internal standard.

#### 3.2.2. Linearity and the Lower Limit of Quantification

Linearity was achieved in a range of 25.5–1020 ng/mL for 6-MP and 6-MMP and a range of 51–1020 ng/mL for 6-TGMP. The linear regression of each calibration curve result was consistent with correlation coefficients (*R*^2^) > 0.9940, 0.9878, and 0.9882, respectively. LLOQ concentrations of 6-MP and 6-MMP were both 25.5 ng/mL, and that of 6-TGMP was 51 ng/mL. 

#### 3.2.3. Accuracy and Precision

Accuracy and precision were determined at four concentrations, the LLOQ, low-quality control (LQC), medium-quality control (MQC), and high-quality control (HQC). The procedure was evaluated by analyzing samples within-run and between-run. Both parameters should be ≤15% for the QC samples and should be ≤20% for the LLOQ. Within-run and between-run precision and accuracy were always less than 15% for the QC samples and less than 20% for the LLOQ.

#### 3.2.4. The Matrix Effect

The matrix effect was assessed using at least six lots of blank matrix from individual donors. The result indicated that ion suppression affected the ionization in LC-MS/MS. Ion suppression or enhancement within 10% indicates no interference affecting the ionization of analytes [17]. In this analysis with DBS, most target compounds showed ion suppression values above 10%, indicating that DBS card components had considerable effects on the ionization of the analytes. Accuracy was always less than 15% for the analytes and internal standard.

#### 3.2.5. Dilution Integrity

The dilution integrity test aimed to ensure that the dilution of samples did not affect accuracy or precision. Data dilution integrity in ½ and ¼ of the ULOQ was always less than 15% for the analytes. 

#### 3.2.6. Stability

The storage stability of analytes in DBS was evaluated to determine whether degradation occurred during long-term storage. Stability was determined by analyzing QC samples stored at room temperature over a period of 30, 60, and 90 days. The QC samples did not show degradation after 90 days of storage at −20 °C, which was a deviation from freshly prepared stock. The analytes were stable for 90 days with a differential from −1.45 to 11.44%, respectively.

#### 3.2.7. Analysis of Study Samples

All samples were obtained at least 6 days and 8 h after the preceding 6-MP dose (75 mg/m^2^/day). The rule of thumb is that a steady state will be achieved after five half-life periods (97% of steady state achieved) [1]. Monitoring of 6-MP levels and its metabolites in the blood is necessary to obtain appropriate information regarding the availability of 6-MP metabolites in both active and inactive metabolites in individual therapy. In the case of chemotherapy, clinical research not only relates to drug efficacy alone, but also reduces the side effects associated with chemotherapy, which is the focus of several studies. This approach is particularly important for ALL patients receiving 6-MP therapy because this drug has therapeutic and hepatotoxicity yield variability [2,6]. The concentration of 6-TGN in plasma was less than 3% compared to whole blood, and 6-MMP was found in plasma samples with levels no higher than 5% compared with those measured in whole blood [18].

**Figure 3:**
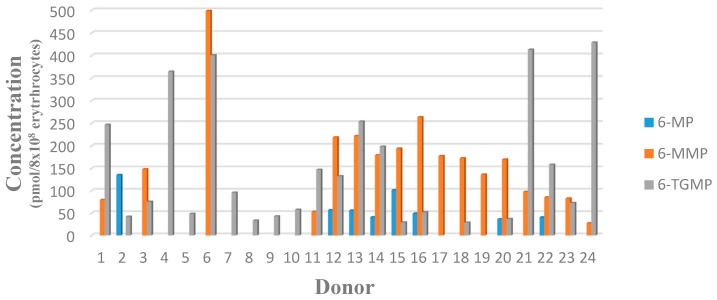
Concentrations of 6-mercaptopurine (6-MP), 6-methylmercaptopurine (6-MMP), and 6-thioguanosine-5′-monophosphate (6-TGMP) in dried blood spots (DBS) from 24 ALL patients.

The 6-TGMP, as an active metabolite, was found in 22 of 24 patient samples, with the lowest level being 51 ng/mL or 29 pmol/8 × 10^8^ erythrocytes (assuming 0.04 × 10^9^ erythrocytes per 100 µL of packed erythrocytes) from Patient SN18, and the highest level being 429 pmol/8 × 10^8^ erythrocytes from Patient SN24. LLOQ concentrations were not available in two patients’ samples. The lowest level of 6-MMP, as an inactive metabolite, was 28 pmol/8 × 10^8^ erythrocytes from Patient SN01, and the highest level was 499 pmol/8 × 10^8^ erythrocytes from Patient SN04. LLOQ concentrations were not available in seven patients’ samples. Results are shown in Table 1 and Figure 3.

Concentrations of 6-TGMP in patients varied. A percentage of 22.7% of patients had concentrations in the therapeutic range, which indicates that the treatment was effective, while 73.7% had concentrations below the therapeutic range, which indicates that a dose adjustment is needed. These latter patients, thus, show a risk of relapse [19]. Concentrations of 6-MMP in 17 patients were below hepatotoxicity thresholds. The value of monitoring 6-mercaptopurine therapy with erythrocyte 6-TGN and 6-MMP concentrations has, thus, been established, and therapeutic thresholds have been associated with increased likelihood efficacy (6-TGN: 235 pmol/8 × 10^8^ erythrocytes), increased risk for leukopenia (6-TGN: 450 pmol/8 × 10^8^ erythrocytes), and increased risk for hepatotoxicity (6-MMP: >5700 pmol/8 × 10^8^ erythrocytes) [20,21]. 

Individual drug therapy trials using this test protocol should provide valuable information for medical practitioners with regard to improving the effectiveness of ALL therapy and reducing the associated side effects. Based on these result, 6-MP dosages can be adjusted so as to maintain remission and prevent toxicity. The DBS method using LC-MS/MS can be used to adjust the 6-mercaptopurin dose of ALL patients in efforts to achieve the maximum therapeutic benefit with minimum side effects.

## 4. Conclusions

The DBS method was utilized to quantitatively analyze 6-MP, 6-MMP, and 6-TGMP concentrations in 24 ALL patients. A total of 27.3% of patients had concentrations in the therapeutic range, which indicates that the treatment is effective, while 73.7% of patients had concentrations below the therapeutic range, which indicates that a treatment dose adjustment is needed. The 6-MMP metabolite levels obtained from 17 patients were below hepatotoxicity thresholds. This method was, thus, effectively utilized for therapeutic drug monitoring.

## Figures and Tables

**Table 1:** Drug concentrations in dried blood spots (DBS) from ALL patients.

Donor	Mean Measured Concentration ± SD (*n* = 2)					
	ng/mL			Pmol/8 × 10^8^ Erythrocytes		
	6-MP	6-MMP	6-TGMP	6-MP	6-MMP	6-TGMP
SN01	N/A	56.5 ± 3.87	401.19 ± 19.53	N/A	79.38	246.89
SN02	100.27 ± 14.81	N/A	78.59 ± 1.31	135.57	N/A	42.42
SN03	N/A	N/A	148.45 ± 6.72	N/A	N/A	75.72
SN04	N/A	315.09 ± 3.49	668.32 ± 9.19	N/A	392.38	364.52
SN05	N/A	N/A	87.34 ± 3.87	N/A	N/A	48.51
SN06	N/A	465.85 ± 9.14	854.01 ± 8.24	N/A	498.90	400.58
SN07	N/A	N/A	182.02 ± 12.94	N/A	N/A	96.05
SN08	N/A	N/A	182.02 ± 0.72	N/A	N/A	96.05
SN09	N/A	N/A	92.20 ± 5.52	N/A	N/A	42.86
SN10	N/A	N/A	112.58 ± 5.53	N/A	N/A	57.82
SN11	N/A	50.52 ± 3.00	319.88 ± 15.89	N/A	53.04	147.11
SN12	37.86 ± 0.59	158.98 ± 0.26	219.99 ± 17.15	56.93	218.90	132.67
SN13	38.23 ± 0.74	164.96 ± 5.40	431.25 ± 12.99	56.35	222.69	254.98
SN14	33.13 ± 14.99	158.75 ± 12.62	400.73 ± 19.02	40.93	179.59	198.56
SN15	92.11 ± 14.98	192.73 ± 4.31	66.19 ± 13.23	101.42	194.32	29.23
SN16	37.18 ± 1.50	217.26 ± 14.41	107.39 ± 0.53	49.28	263.73	57.09
SN17	N/A	157.15 ± 2.24	N/A	N/A	177.78	N/A
SN18	N/A	133.92 ± 8.89	51.13 ± 3.32	N/A	172.75	28.86
SN19	N/A	129.91 ± 0.43	N/A	N/A	136.39	N/A
SN20	30.30 ± 7.88	153.94 ± 9.50	76.83 ± 8.60	36.57	170.16	37.19
SN21	N/A	83.97 ± 1.27	810.37 ± 13.16	N/A	97.74	413.17
SN22	30.94 ± 5.49	70.91 ± 2.40	300.27 ± 10.15	40.71	85.43	158.45
SN23	N/A	64.23 ± 0.26	129.14 ± 11.45	N/A	82.76	72.88
SN24	N/A	24.06 ± 5.91	853.36 ± 17.40	N/A	27.61	428.88

SN01: subject number 1; 6-MP: 6-mercaptopurine; 6-MMP: 6-methylmercaptopurine; 6-TGMP: 6-thioguanosine-5′-monophosphate; N/A: not available.
